# The Impurity Removal and Comprehensive Utilization of Phosphogypsum: A Review

**DOI:** 10.3390/ma17092067

**Published:** 2024-04-28

**Authors:** Qingjun Guan, Zhuang Wang, Fujia Zhou, Weijian Yu, Zhigang Yin, Zhenyue Zhang, Ru’an Chi, Juncheng Zhou

**Affiliations:** 1 School of Resource Environment and Safety Engineering, Hunan University of Science and Technology, Xiangtan 411201, China; qshq78@163.com (Z.W.); kunjia2012@foxmail.com (F.Z.); ywjlah@163.com (W.Y.); 2 Hunan Province Key Laboratory of Coal Resources Clean-Utilization and Mine Environment Protection, Xiangtan 411201, China; 3 Lithium Resources and Lithium Materials Key Laboratory of Sichuan Province, Tianqi Lithium Corporation, Chengdu 610213, China; 4 School of Xingfa Mining Engineering, Wuhan Institute of Technology, Wuhan 430073, China; zyzxmu@wit.edu.cn; 5 Hubei Three Gorges Laboratory, Yichang 443007, China; rac@wit.edu.cn; 6 School of Mechatronics Engineering, Chengdu University of Technology, Chengdu 610059, China; 18782532617@163.com

**Keywords:** phosphogypsum, impurity removal, utilization, rare earth elements

## Abstract

Phosphogypsum (PG), a byproduct during the phosphoric acid production process, also known as the wet process, contains complex and diverse impurities, resulting in low utilization and considerable accumulation. This leads to a massive waste of land resources and a series of environmental pollution problems. Given the current urgent ecological and environmental situation, developing impurity removal processes with low energy consumption and high efficiency, exploring valuable resource recovery, preparing high value-added PG products, and broadening the comprehensive utilization ways of PG are significant strategies to promote the sustainable consumption of PG and sustainable development of the phosphorus chemical industry. This review comprehensively summarizes the advantages and disadvantages of existing PG impurity removal and utilization technologies and probes into the future development direction, which provides references and ideas for subsequent PG research.

## 1. Introduction

**Figure 1:**
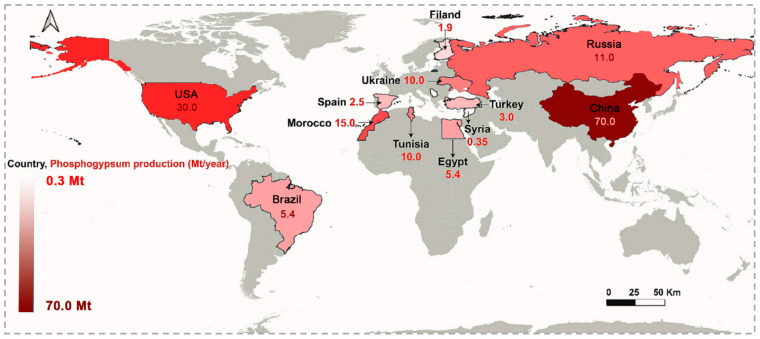
Annual production of PG in various countries around the world.

In the 21st century, with the rapid development of modern agriculture and industry, the phosphorus chemical industry has achieved unprecedented growth, resulting in huge amounts of PG. Its main component is dihydrate gypsum (CaSO_4_·2H_2_O), and it also contains a large amount of phosphorus, fluorine, organic matter, and other impurities. A total of 4–5 tons of PG are produced for every 1 ton of phosphoric acid product [1]. Figure 1 shows the approximate annual production of PG in various countries around the world at present [2]. The growth rate of PG is about 1.7 × 10^8^ tons per year, and only 15% of globally produced PG is valorized [3]. By the end of 2020, the global PG solid waste has reached 7 × 10^9^ tons, of which China’s stockpile is about 8.2 × 10^8^ tons and the growth rate is 7 × 10^7^ tons per year [4,5]. Most PG is stored or landfilled, occupying large amounts of land and easily polluting the surrounding atmosphere, water, and soil [6]. There are two main reasons for the low utilization rate of PG; primarily, large amounts of impurities, especially the high radioactivity levels caused by radium and other radionuclides [7], seriously limited the quality and utilization of PG products, followed by the low value-added products that significantly restricted the sale radius. Therefore, reducing the impurity content and increasing the added value of PG products is a crucial way to solve the problem of PG accumulation [8,9,10].

In this review, we systematically summarize the impurity removal methods and reuse approaches of PG and compare the advantages and disadvantages of various methods. On this basis, some innovative ideas and strategies for the effective consumption of PG were put forward.

### 1.1. Physical and Chemical Properties of PG

PG is usually a yellowish-white, light grayish-white, or dark gray fine powdery particle that is acidic (pH 2.37~5.33), whose micromorphology is mainly of a rhombic flake [11]. The main component of PG is CaSO_4_·2H_2_O (mainly > 80 wt%). The chemical compositions of PG in different regions are shown in Table 1. In addition to the major components listed in Table 1, PG generally contains radioactive elements (Table 2) and rare earth elements as shown in Section 3.3.

PG is radioactive due to the presence of naturally occurring uranium and thorium, as well as their daughter nuclides radium, radon, polonium, etc. These elements are concentrated in PG during the digestion of phosphate ores with sulfuric acid [12]. Thus, it can be considered a technologically enhanced naturally occurring radioactive material (TENORM) [13]. This is one of the major obstacles to the recycling utilization of PG.

**Table 1:** Chemical compositions of PG in different studies.

	Constituents (wt%)	CaO	SO_3_	SiO_2_	P_2_O_5_	F^-^	Al_2_O_3_	Fe_2_O_3_	MgO	LOI	Origin of PG
Ref.											
Değirmenci [14]		32.04	44.67	0.44	0.50	0.79	0.88	0.32	-	21.06	Bandirma, Turkey
Yang, et al. [15]		29.05	42.19	1.25	3.50	-	0.43	0.21	-	19.48	Guizhou, China
Zhao, et al. [16]		32.12	46.02	5.94	1.39	0.06	0.5	1.54	0.3	-	Sichuan, China
Ajam, et al. [17]		32.8	44.4	1.37	1.69	0.55	0.11	0.03	0.01	22.3	Sfax, Tunisia
El Zrelli, et al. [18]		37.18	37.5	1.03	1.11	1.66	0.06	0.13	0.07	-	Gulf of Gabes, Tunisia
Gaidučis, et al. [19]		34.35	51.11	4.35	2.33	0.50	0.24	0.06	0.72	6.34	Morocco and Kovdor
Ennaciri, et al. [20]		31.71	43.4	0.74	1.2	1.1	0.26	0.07	0.08	20.8	Jorf Lasfar, Morocco
Akfas, et al. [21]		39.3	54.6	0.12	0.88	0.61	0.05	-	-	-	Jorf Lasfar, Morocco
Gaidučis, et al. [19]		37.16	52.21	-	1.84	0.49	0.15	0.12	1.43	6.61	Kovdor, Russian
Kapustin, et al. [22]		31	44.27	0.87	0.45	-	0.93	0.2	0.03	20.73	Sverdlovsk region, Russia
Kadirova, et al. [23]		36.48	49.89	1.89	0.44	-	0.08	-	-	11.22	Tashkent region, Uzbekistan
Rashad [24]		32.14	34.51	8.82	1.72	0.8	0.29	0.35	0.09	21	Cairo, Egypt
Kandil, et al. [25]		28.31	40.45	8.92	1.98	0.26	0.17	0.31	0.21	19.71	Fayoum City, Egypt
Nguyen and Le Vu [26]		28.07	42.5	2.99	2.93	-	0.56	0.87	0.69	19.92	Vietnam
Jancev, et al. [27]		28.8	41.7	0.47	8.22	0.4	0.12	0.08	0.01	19.83	Macedonia
Yelatontsev and Mukhachev [28]		34.1	53.3	3.7	0.5	-	1.9	0.2	2.1	-	Ukraine

LOI: Loss on ignition.

**Table 2:** Radioactive activity concentration (Bq/kg) of PG in different regions.

	Radioactive Elements	^226^Ra	^228^Ra	^238^U	^235^U	^210^Pb	^214^Pb	^232^Th	^234^Th	^40^K	Origin of PG
Ref.											
Değirmenci [14]		22		9				1		11	Bandirma, Turkey
Reguigui, et al. [29]		215		47				15		-	Sfax, Tunisia
Mahjoubi, et al. [30]		350		-				-		-	Sfax, Tunisia
Papastefanou [31]		261		-				-		-	Greece, Togo
Rutherford, et al. [32]		451–500		510				10		-	Australia
Rutherford, et al. [32]		1120		130				3.7		-	Florida, USA
Azouazi, et al. [33]		1420		-				-		-	Morocco
Bigu, et al. [34]		411		134				19		-	Egypt
Aguado, et al. [35]		727		-				-		-	Spain
Luca, et al. [36]		100	-	-	2.9	150	72	-	47	-	IAEA
Papastefanou, et al. [37]		585	3.3	19	-	430	-	3.3	-	13	Thessaloniki, Greece
Msila, et al. [38]		109	404	31	1	135	-	189	-	<100	South Africa
Da Conceicao and Bonotto [39]		269–280	-	298–310	298–310	-	-	185–206	-	60–63	Tapira city, Brazil
Rentería-Villalobos, et al. [40]		785	-	100	-	827	-	-	-	-	Huelva, Spain
Rutherford, et al. [41]		610	-	1.5	-	560	-	3.5	-	-	Togo
Kuzmanović, et al. [42]		656	-					3.1		101	Serbia
Gezer, et al. [43]		436						9		13	Turkey
Alam, et al. [44]		234						21		108	Bangladesh
Mourad, et al. [45]		596						6		2	Nile Delta, Egypt
Al-Jundi, et al. [46]		376						4		40	Jordan
Barescut, et al. [47]		618						8.5		24.1	Korea
Kovler, et al. [48]		747						14		63	Israel

### 1.2. Impurities in PG

Table 3 shows the main impurities contained in PG and their adverse effects.

## 2. Purification

According to the impurity removal mechanism, the impurity removal technology of PG mainly includes physical, chemical, flotation, recrystallization, microbial, and heat treatment methods. The physical methods include water washing, screening, magnetic separation, cyclone classification, etc. The chemical methods mainly include acid leaching, alkali leaching, and neutralization.

### 2.1. Physical Methods

Physical methods are based on the differences in physical properties between gypsum and impurities, such as solubility, particle size, magnetism, etc., through washing, cyclone classification, sieving, magnetic separation, and other processes to remove impurities from PG [53,54,55,56].

#### 2.1.1. Washing

The washing method is to mix PG and water according to a certain solid/liquid ratio so that the water-soluble impurities are dissolved in the water and then removed by solid/liquid separation. Zhang et al. [57] found that after three-stage countercurrent washing with the solid/liquid ratio of 0.5, the soluble phosphorus content in PG was reduced from 0.330% to 0.087% with the removal rate of 78.81%, and the soluble fluorine content was reduced from 0.250% to 0.018% with the removal rate of 89.94%. The pH was 5.58 after washing. However, the process required 2 tons of water for every 1.02 tons of PG and produced large amounts of acidic wastewater, which inevitably raised the cost of subsequent treatment. In addition, water washing was only successful in eliminating impurities adsorbed on the crystal surface, while the majority of lattice-incorporated contaminants remained [58,59,60].

#### 2.1.2. Cyclone Classification

**Figure 2:**
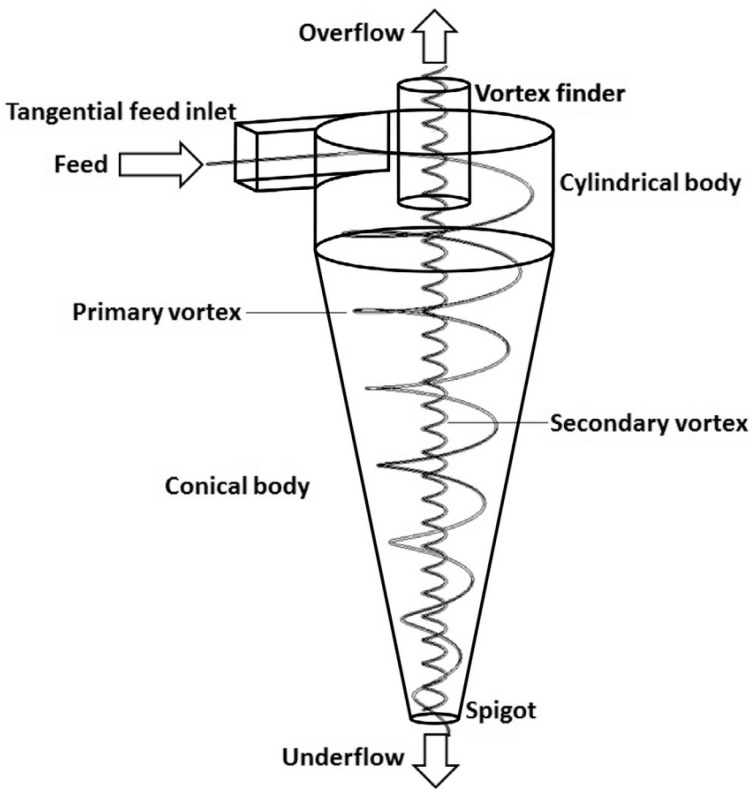
Schematic diagram of the hydrocyclone.

During the cyclone classification process, the slurry is pumped into the cyclone tangentially from the cyclone’s upper cylinder under pressure. Then, it makes a circular motion under the restriction of the cylindrical wall. The particles with different sizes show different motion states due to the different inertial centrifugal forces: the coarse particles settle out and exit at the “underflow” and the fines remain in suspension and exit out the “overflow”, as shown in Figure 2. During the circular movement of the particles in the cyclone, part of the impurities attached to the surface of the gypsum crystal is washed into the liquid phase and removed through solid/liquid separation. The crystalline phosphorus, linked to the fine-grained PG, can be eliminated by classification. The impurity removal efficiency can be regulated by adjusting the operation parameters, such as the settling mouth’s diameter and the feed’s flow rate [61].

Tan et al. [62] explored the impurity removal effect of cyclones on PG. As shown in Figure 2, the classification effect was noticeable. As it can be noticed in Table 4, the underflow products contained fewer impurities than the overflow products, and the hydrocyclone with a settling mouth diameter of 18 mm had a better impurity removal effect than that with a settling mouth diameter of 22 mm. However, the removal efficiency was still low. Therefore, the cyclone was generally used in pretreatment [63].

#### 2.1.3. Sieving

If impurities are concentrated in PG only with a certain particle size range, the impurities would be effectively eliminated by sieving. In a study of Moroccan PG, it was observed that impurities such as fluorine, silicon, and sodium were significantly enriched in the particle size fractions exceeding 170 μm. Conversely, organic substances and co-crystalline phosphorus were mainly enriched in the particle size fractions below 25 μm. This approach recovered nearly 75% of PG in a purified state [64].

Additionally, other physical methods, such as milling and ultrasonic treatment, are used to mechanically fracture the gypsum crystals into smaller particles, which will partly release the impurities from the crystal lattice and thus eliminate the impurities from PG to a larger extent combined with other impurity removal methods [64,65,66].

### 2.2. Chemical Methods

The chemical method, mainly including acid/alkali leaching and lime neutralization, removes the impurities from PG or reduces their impact by converting them into easily separable or relatively stable substances [67,68,69].

#### 2.2.1. Acid/Alkali Leaching

The method includes treating PG with mineral acid (such as sulfuric acid, hydrochloric acid, or phosphoric acid), organic acid (such as citric acid, oxalic acid, boric acid, and malic acid), ammonium sulphate, ammonium hydroxide, or their mixed solutions [59,64,70,71].

Liu et al. [72] found that the whiteness of PG increased from 55.3% to 91% after stirring and undergoing a reaction process for 5 min at 80 ℃ and with a solid/liquid ratio of 1:25 using 3 mol/L sulfuric acid as the leaching agent. This method could effectively remove metal impurities and most phosphorus and fluorine in PG. Singh et al. realized the effective purification of PG by treating it with citric acid and ammonium hydroxide, respectively [70,73]. The purified PG could be an additive instead of mineral gypsum in manufacturing ordinary Portland cement. Yet, these studies are not proficient in completely eliminating the impurities integrated into the gypsum crystal lattice. For the intercrystalline phosphate impurities, Cai et al. [74] illustrated that oxalic acid could eliminate part of co-crystalline phosphorus impurities by disrupting a portion of the gypsum crystal structure, resulting in the phosphorus leaching efficiency of 77.7% with the use of 1% oxalic acid. Although this method is easy to operate, it often requires a large amount of solution, which inevitably faces the problem of subsequent leachate treatment [75,76]. In addition, this method only removes surface-adsorbed impurities, with little effect on those encapsulated.

#### 2.2.2. Neutralization

Neutralization with lime or other basic materials was often used to make impurities in PG ineffective [77,78]. Li et al. [79] found that under the condition of a lime dosage of 0.4% and an aging time of 12h, the soluble phosphorus content in PG decreased from 0.14% to 0.01% with a removal rate of 93.27%, and the soluble fluorine content decreased from 0.02% to 0.012% with a removal rate of only 29.07%. According to the neutralization reactions (1) and (2), the removal process of soluble phosphorus was not affected by pH in the system, but the formation of CaF_2_ would be inhibited when pH increased, which prevented the neutralization of the soluble fluorine [80]. Therefore, this method is more suitable for the treatment of PG with low soluble fluorine content. In addition, this method cannot eliminate the effects of the intercrystalline phosphorus.

(1) $$P_{2}O_{5}+3H_{2}O+3CaO=3{Ca}_{2}{\left(PO_{4}\right)}_{2}↓+3H_{2}O$$

(2) $$2F^{-}+CaO+H_{2}O=CaF_{2}↓+2{OH}^{-}$$

### 2.3. Flotation Method

**Figure 3:**
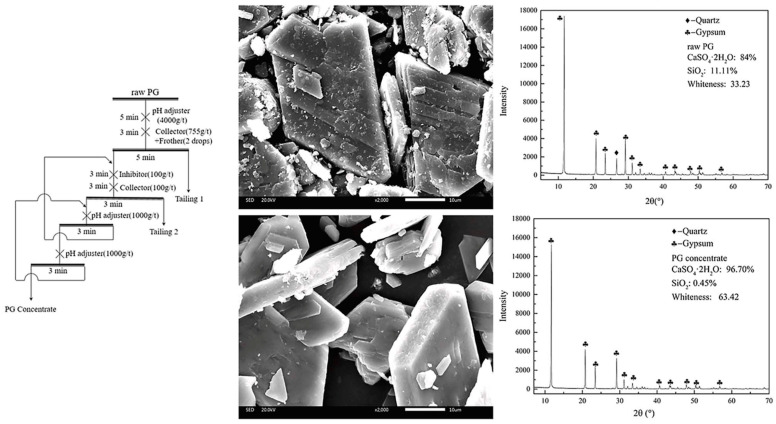
The closed-circuit flotation process and changes in PG before and after flotation [83].

Flotation mainly changes the floatability between different substances by adding flotation reagents to the PG slurry, thereby separating gypsum and impurities [81]. Wang et al. [82] used methyl isobutyl carbinol (MIBC) as the foaming and collecting agents to remove organic matter and mineral mud from PG by reverse flotation and then used dodecyl amine as the collector to recover gypsum by direct flotation. The total phosphorus content of PG obtained was reduced from 1.78% to 0.92%, the content of CaSO_4_·2H_2_O reached 96.5%, and the concentrate yield was 65%. Ji et al. [83] also adopted the idea of “reverse-direct flotation” to remove impurities from a typical high-silica PG waste. The organic substances and fine slimes were eliminated through reverse flotation, followed by the removal of silica impurities via direct flotation. Through the closed-circuit flotation process depicted in Figure 3, the whiteness of the PG concentrate is enhanced from 33.23 to 63.42, and the purity rises from 83.90% to 96.70%, achieving a gypsum recovery rate of 85%.

The flotation method has two obvious flaws. One is that intercrystalline impurities cannot be eliminated, and the other is that the whiteness of the product is not high, which limits its application fields [84].

### 2.4. Recrystallization Method

**Figure 4:**
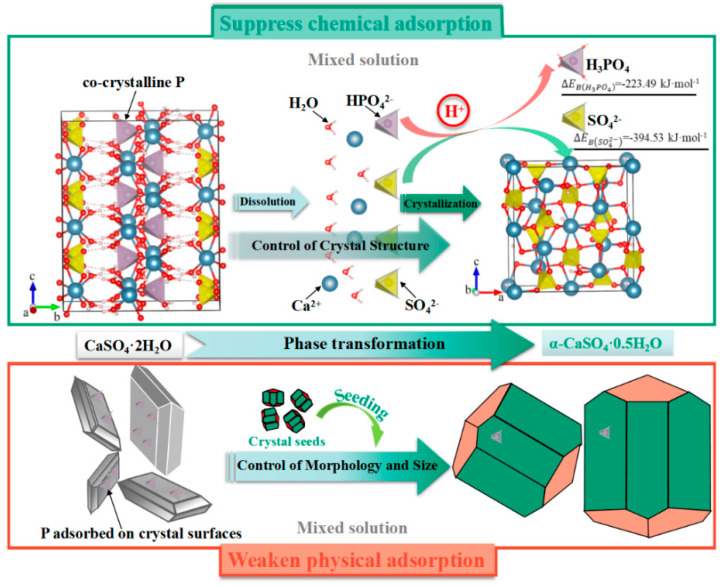
Mechanism diagram of deep phosphorus removal from PG based on crystal regulation induced by seeding [88].

The recrystallization method can fully release the impurities incorporated into the gypsum lattice through PG’s dissolution–recrystallization process, transforming into α-CaSO_4_·0.5H_2_O or CaSO_4_ in a solution system [85,86]. Meanwhile, a specific type of regulator is added to control the form of released impurities to inhibit them from entering the recrystallized product again, ultimately achieving efficient separation of impurities. Our research team has undertaken extensive and valuable investigations in this domain. We achieved the efficient removal of intercrystalline phosphorus (P) impurities and the simultaneous production of high strength gypsum from PG by phase transformation under mild conditions in salt–acid mixed solutions (such as NaCl-HCl, Na_2_SO_4_-H_2_SO_4_ and CaCl_2_-HCl solution) [87,88,89,90]. The co-crystalline P was completely liberated during the dissolution of phase transition from PG to α-CaSO_4_·0.5H_2_O in salt solutions, which was essential for the effective removal of co-crystalline phosphorus impurities. Adding mineral acids as the regulators to salt solutions dissolved the released P impurities and transformed them into the species (H_3_PO_4_) with less similarity to SO_4_^2−^, which prevented the recombination of the released P impurities with gypsum. After regulating the phase transformation and P species in salt–acid mixed solutions, the P removal rate reached more than 80%. Moreover, α-CaSO_4_·0.5H_2_O shape and size were controlled by seeding in the mixed solutions. The formation of large-grained short-columnar α-CaSO_4_·0.5H_2_O suppressed the physical adsorption of P on the product surface and further increased the removal rate to above 95%. Furthermore, this large-grained short-columnar α-CaSO_4_·0.5H_2_O exhibited substantial mechanical strength, making it a high-value-added gypsum product. The mechanism diagram is depicted in Figure 4.

In addition, some persulfate salts such as K_2_S_2_O_8_ could also be used as regulators, which were activated under hydrothermal conditions to produce sulfate radicals, oxidizing and degrading organic black impurities in gypsum, and promoting the whiteness of gypsum [91,92,93].

### 2.5. Microbiological Method

**Figure 5:**
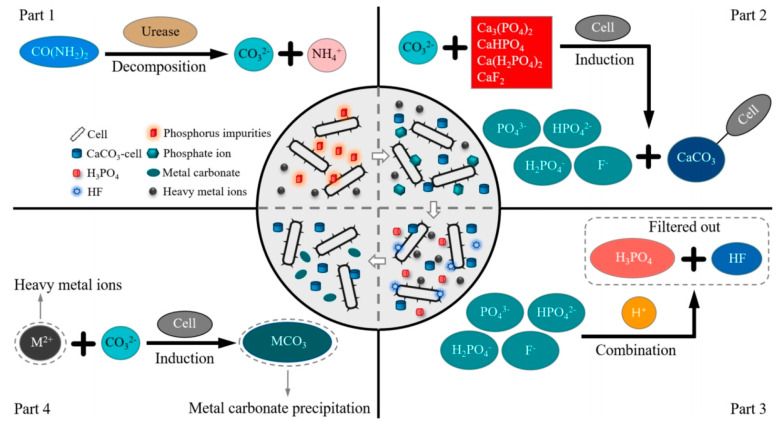
Mechanism diagram of MICP for the removal of the impurities and heavy metals [96].

**Figure 6:**
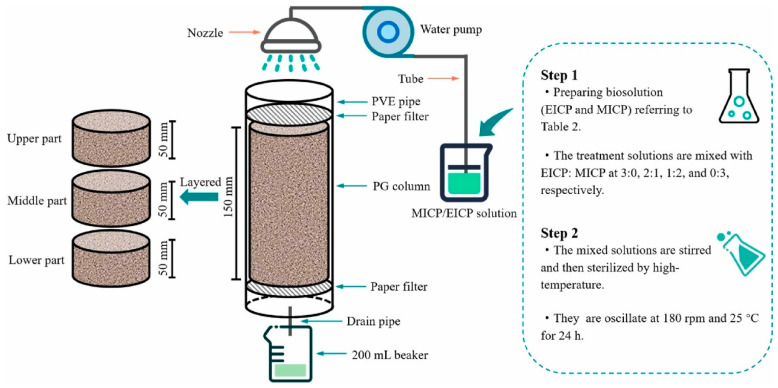
Design diagram of MICP/EICP treatment for PG [98].

Microbially induced carbonate precipitation (MICP) as shown in Figure 5 is extensively recognized as an eco-friendly impurity removal technology [94]. This method removes impurities from PG by the biomineralization of Ca^2+^ in impurities with CO_3_^2−^ produced by microbial metabolism [95]. Xiang et al. [96] effectively eliminated 74–77% of phosphorus and fluorine impurities and 50–70% of heavy metals in PG using the MICP technology, and the processed PG contained a substantial quantity of calcium carbonate, enhancing the early strength of the cement paste blended with PG by 15–20%. Nonetheless, the low pH of PG makes it challenging to directly utilize many bacilli for PG’s processing. Thus, acidophilic bacteria were introduced to treat PG, but their low mineralization rate led to low removal of impurities. To solve this problem, Xiang et al. introduced a novel technique employing an enzyme-induced carbonate precipitation (EICP)-modified acidophilus bacteria solution to eliminate phosphorus and fluorine from PG, as depicted in Figure 6 [97,98]. The removal efficiency of P and F reached the highest, ranging from 72.87 to 74.92%, at the ratio of MICP to EICP of 2:1. The robust acid resistance of the urease enzyme and acidophilic bacteria enhanced their growth and activity, leading to an approximately 22% increase in the biomineralization rate. Furthermore, the MICP/EICP treatment was 30% more cost-effective than the conventional binder treatment.

### 2.6. Heat Treatment Method

The heat treatment, also known as the calcination method, involves the volatilization of fluoride and organic substances at elevated temperatures, transforming various forms of calcium phosphate into pyrophosphate [99,100]. This approach boasts rapid processing speed but incurs high energy consumption.

**Figure 7:**
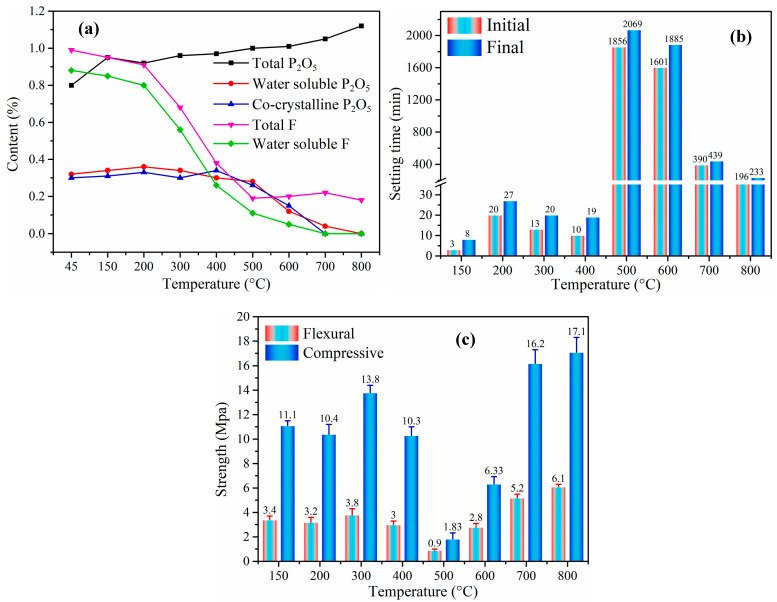
P_2_O_5_ and F content under different temperatures (**a**). The setting time of CPG under different temperatures (**b**). The compressive and flexural strength of HPG under different temperatures (**c**) [102].

Ba et al. [101] proposed an improved calcination process using a two-step method. The characteristics of this process were that PG added with neutralizer was suspended in the system, thus obtaining a larger heat exchange area and more thorough calcination. Simultaneously, the waste heat could convert the dihydrate gypsum into bassanite. Cao et al. [102] explored the impact of impurities on the characteristics of gypsum plaster at increasing calcination temperatures. As depicted in Figure 7, detrimental impurities exhibited minimal alteration at low calcination temperatures (150–400 °C), contributing to diminished mechanical properties despite a higher degree of hydration. Nevertheless, the calcination at elevated temperatures transformed detrimental impurities into inert, insoluble compounds such as calcium pyrophosphate (Ca_2_P_2_O_7_), calcium metaphosphate (Ca(PO_3_)_2_), and calcium fluoride (CaF_2_). At the optimal calcination temperature of 800 °C, gypsum plaster exhibited rapid setting and maximum strength, featuring a dense microstructure and low hydration degree attributed to the substantial formation of Na_2_SO_4_ and nucleation sites.

In summary, scholars have developed various PG impurity removal techniques, each of which has pros and cons, as shown in Table 5. Some low-cost impurity removal methods, such as washing, cyclone classification, neutralization, and flotation, have been used in practical production. However, these methods cannot eliminate the intercrystalline impurities, which limits the application field and value of PG products. If efficient removal of impurities from PG is to be achieved, such as eliminating impurities using the recrystallization method, the cost will also increase. However, if these costly efficient impurity purification processes could be integrated in the production chains of high-value gypsum-based products, they would become more applicable and affordable.

## 3. Utilization

**Figure 8:**
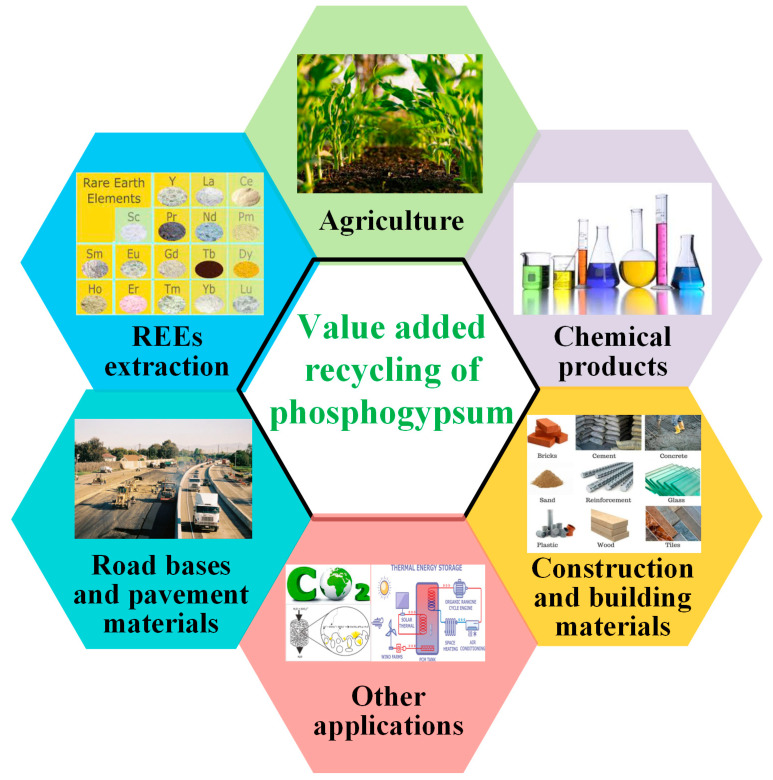
Potential valorization pathways of PG.

The reuse of PG is the necessary way to solve its accumulation. Over the years, researchers have conducted various studies on the comprehensive utilization of PG, mainly including applications in construction and building materials, extraction of rare earth elements (REEs), preparation of chemical products, applications in agriculture, and other applications, as depicted in Figure 8.

### 3.1. Construction and Building Materials

Due to its calcium sulfate nature, PG serves as a replacement for natural gypsum in the manufacturing of cement, wallboard, road base and pavement material, ferrocement panels, plasterboard panels, partition blocks, plaster, artificial stone, glass ceramics, and various other building materials [103,104,105]. Using PG in construction materials instead of natural gypsum offers numerous potential advantages. It is frequently more easily accessible and cost-effective compared to natural gypsum, contributing to a reduction in the environmental footprint of the construction industry by decreasing the need for mined gypsum [2]. Nevertheless, incorporating PG may exert a specific influence on the properties of the matrix, including workability, setting time, mechanical strength, and unit weight [106,107,108].

The addition of PG would decline the workability of the matrix. For example, Yang et al. [109] formulated a self-leveling mortar blend comprising 20% cement, 40% PG, and 40% natural sand. Substituting natural sand with PG resulted in a reduction in the initial workability. Buhari and Raju [110] illustrated a decrease in the workability of concrete mixtures when incorporating PG as a substitute for cement at levels of 5%, 7.5%, 10%, 12.5%, and 15%. Sindhuja et al. [111] also illustrated a reduction in the workability of concrete mixtures when using PG as a substitute for cement at levels of 10%, 20%, and 30%. The decline in the workability was probably ascribed to the low density of PG compared to those of natural sand or cement. This is considered as one of the defects of using PG in construction and building materials. The introduction of PG led to longer initial and final setting time of the mixture. The increment in setting time was increased with increasing PG content. Nigade and Bagade [112] demonstrated a 220%, 442.65%, 597.14%, 722.85%, and 728.57% increase in the initial setting time of concrete mixtures when incorporating 5%, 10%, 15%, 20%, and 25% PG as a substitute for cement, respectively. Meanwhile, the final setting time was extended by 29.8%, 44%, 102.13%, 106.4%, and 107.45%, respectively. Additionally, The considerably prolonged retarder effect of PG may arise from the creation of protective coatings, such as CaF_2_ and Ca_3_(PO_4_)_2_, formed by inactive elements produced by PO_4_^3-^ and F^-^ on the surface of cement particles during mixing [113].

Generally, the incorporation of untreated PG in the matrix decreased its mechanical strength, and the decline in strength became more pronounced with higher PG content, which was ascribed to the impurities in PG. Deepak et al. [114] found that incorporating 10%, 20%, and 30% PG as a substitute for cement in concretes resulted in a decrease of 1.1%, 37.84%, and 44.32% in the compressive strength at 7 days, respectively. Similarly, the compressive strength at 28 days declined by 1.25%, 38.28%, and 44.44%, respectively. Nevertheless, in the majority of instances, incorporating treated or calcined PG up to a certain level in the matrix improved its mechanical strength or alleviated the strength degradation caused by the untreated one [115]. As shown in Table 6, the advantages of including PG in the matrix are reducing density, increasing chemical resistance, increasing freeze/thaw resistance, and increasing fire resistance. On the other hand, the disadvantages of including PG in the matrix are decreasing workability, decreasing mechanical strength, decreasing abrasion resistance, increasing drying shrinkage, increasing soundness expansion, and increasing thermal conductivity. Increasing setting time can be considered as an advantage or a drawback according to the application which PG is used for. Some of the mentioned disadvantages of using PG can be alleviated by applying the purification technology as reported in the Section 2 of this article. It is worth mentioning that despite the adverse impact of PG on certain properties of the matrix, using PG as a construction and building material is an essential way to consume PG [116,117,118].

So far, the described investigations have reviewed the utilization of PG in building products not as the main binding material, but as an additive to cement or as a supplementary cementitious material. However, PG can also be employed with more ambition as the main binding material in Portland cement-free building products. For instance, Zhou et al. [119] proposed a novel “Two-step Hydration Process” for preparing PG non-fired bricks of a high 7-day compressive strength of 29 MPa, using no cement and a low pressure of 10 MPa in press-forming. The optimal formulation comprised 75.0% of PG, 23.47% of river sand and 1.53% of hydrated lime, and the water incorporation was 22% of all the above solids. The corresponding water absorption, weight loss, and compressive strength after 15 freezing–thawing cycles of as-prepared 7-day bricks were 10.19%, 1.1%, and 23 MPa, respectively, which fully met the quality requirements of the highest MU25 grade in the Chinese standard (JC/T422-2007).

### 3.2. Road Bases and Pavement Materials

Due to large consumption, the utilization of PG in road bases and pavement materials becomes an important way to solve the problem of large PG stockpiles [120].

A mixture of 46.5% sand, 46.5% PG, and 7% cement was prepared, and its mechanical strength and leaching behavior were discussed [121]. The obtained results indicated that the early and final compressive strengths of the stabilized material were higher than those of conventional road subgrade materials, reaching 2200 and 3500 kPa at 28 and 360 days, respectively. The leaching tests demonstrated that almost all contaminants studied were significantly immobilized in the mixture with high pH values. Dutta and Kumar [122] mixed fly ash and lime with PG and produced a desirable road material. It was concluded that the fly ash–lime–PG composite (fly ash + 8% lime + 2% PG) satisfied the unconfined compressive strength, split tensile strength, and slake durability criteria, as well as giving higher bearing ratio values at 28 days of curing. Therefore, the fly ash–lime–PG composite cured for 28 days could be used as a base/subbase course material in road pavements. The experiment results obtained by Mashifana, et al. [123] implied that composites containing 50% PG had an unconfined compressive strength of 1.08 MPa and optimum replacement of 30% of the LFA (lime–fly ash), with slag further improving the strength to 3.2 MPa. The final composites met the requirements of South African Roads (TRH4, 1996) specification and had equivalent strength values as C1–C4 materials, fitting criteria for subbase in terms of strength durability.

Reflection cracks in asphalt pavements, predominantly induced by shrinkage fissures in the subgrade, have emerged as one of the most prevalent issues affecting pavements. In a bid to counteract the shrinkage tendencies inherent in stabilized subgrades, a novel slow-setting, mildly expansive cement was formulated as the road base course binder (RBCB), featuring an experimentally determined optimal composition comprising 45% fly ash, 45% silicate cement clinker, and 10% gypsum [124]. The RBCB exhibited a 28-day expansion rate of 4‰, thereby effectively offsetting cement shrinkage and enhancing volume stability under both wet and dry conditions. Notably, the strength of aggregates stabilized with RBCB surpassed that of P.S 32.5 stabilized aggregates by 15% with equivalent cement content, thereby facilitating a reduction in cement usage by 10–15%. Furthermore, RBCB markedly enhanced the splitting strength of the base material. Relative to conventional cement-stabilized aggregates, RBCB-stabilized aggregates demonstrated a reduced dry shrinkage ratio, resulting in an 82% reduction in reflective cracks within semi-rigid subgrade pavements and a prolonged road service life.

The utilization of PG in highway engineering remains in its nascent phase, primarily characterized by investigations into its impact on the behavior of road bases or pavement materials. However, scant attention has been paid to its durability or its enduring influence on soil and water resources. Hence, there is a pressing need for additional research endeavors to advance the utilization of PG in this field.

Moreover, a key obstacle hindering the application of PG in the construction and building materials or road bases and pavement materials is its radioactivity. The European Commission established a limit gamma emission rate of 1 mSv/year [125]. Usually, this limit is not depicted directly but is evaluated through the radioactivity concentration index *I*, calculated from the activity concentration of radionuclides ^226^Ra, ^232^Th, and ^40^K. A material is considered safe from a radiological perspective when *I* ≤ 1.0, if employed in bulk amounts (slabs, wall blocks, etc.) or *I* ≤ 6.0, if used in superficial applications (wall coatings, ceilings, etc.) [126]. If this condition is not met, the material could be hazardous to human health, so additional investigations must be performed.

### 3.3. Recovery of REEs

The phosphate ore often contains rich REEs, an essential strategic resource. More than 70% of REEs in the phosphate ore end up in the PG during the wet production of phosphoric acid, and thus, PG presents an appealing secondary source for REEs as a significant quantity of them is contained within PG [127,128,129]. The REE content in PG exhibits considerable variation across different regions, ranging from less than 0.01 wt% to as high as 6.8 wt%. Mineral and some organic acids are frequently employed as the lixiviant in laboratory experiments for the extraction of REEs from PG. Of these, sulfuric acid is a preferred agent, given its easy availability and cost-effectiveness. Nevertheless, sulfuric acid solutions under standard laboratory conditions have usually yielded relatively low leaching efficiencies for REEs, typically ranging from 12% to 40% (as shown in Table 7), which might be ascribed to the inefficient diffusion of ions, low solubility of gypsum caused by the common-ion effect, or the formation of sparingly soluble double REE sulfates [129,130,131,132,133].

To intensify the REE leaching from PG, various approaches have been implemented [134,135,136]. In one of our previous reviews [137], we classified these methods into five categories: physically enhanced leaching method (including mechanical activation, increasing the liquid/solid ratio or number of leaching, extending the leaching time, and ultrasonic or microwave treatment), chemically enhanced leaching method (including the resin-in-leach (RIL) process and organic liquid leaching), phase inversion-enhanced leaching method (including carbonation and recrystallization), bioleaching method, and a joint method. Despite much research on extracting REEs from PG, its industrialization remains to be a long way off from reality. Moreover, it is meaningless to only recover REEs from PG owing to their very low content, and it should be coordinated with the high value of PG.

**Table 7:** Part of previous studies on REE leaching from PG [137].

Origin	Country	REO or REE (wt%)	Leaching Conditions					Leaching Efficiency (%)	Ref.
			Leaching Regent	Conc. (M^a^ or wt%^b^)	Temp. (℃)	Time (h)	L/S (mL/g)		
Kola	Russia	0.6	H_2_SO_4_	10–15 ^b^	40	6	2/1	52	Jarosiński et al. [138]
Phosphoric acid plant at Phalaborwa	South Africa	6.8	HNO_3_	2.0 ^a^	20	48	3/1	57	Preston et al. [139]
Abu-Zaabal Company in Cairo	Egypt	0.022	HNO_3_; Ca(NO_3_)_2_	2.0–3.0 ^a^; 0.8 ^a^	25	8	1/1	76	El-Reefy et al. [140]
			HNO_3_	2.0 ^a^				46	
			HCl	4.0 ^a^				30	
			H_2_SO_4_	4.0 ^a^				30	
Private Joint Stock Company ‘Metakhim’, Leningrad Oblast	Russia	0.414	H_2_SO_4_	0.5–4.0 ^b^	25	3025	2/1	57.1–68.2	Lokshin et al. [129]
Dump PG in Kola	Russia	0.45	H_2_SO_4_; HNO_3_	1.0–3.0 ^b^	—	8–12 min	4/1–5/1	85–86.1	Abramov et al. [141]
Synthetic PG in Florida	USA	0.034	H_2_SO_4_; H_3_PO_4_	25 ^b^; 96 ^b^	72	1	20/3	49	Al-Thyabat and Zhang [142]
Abu-Zaabal Company, Cairo	Egypt	0.048	HNO_3_	3.0 ^a^	25	3	2/1	43.3	Ismail et al. [143]
			HCl	2.0 ^a^				11.9	
			H_2_SO_4_	4.0 ^a^				12.5	
Agrium Fertilizer plant in Calgary, Alberta	Canada	0.020	HNO_3_	1.5 ^a^	80	2	8/1	57	Walawalkar et al. [130]
			HCl					51	
			H_2_SO_4_					23	
Mosaic company in Tampa, Florida	USA	0.0218	H_2_SO_4_	5.0 ^a^	50	3.5	4/1	43	Liang et al. [144]
Nutrien Ltd.’s fertilizer operations in Saskatoon, Saskatchewan	Canada	0.0317	HCl	1.5 ^a^	85	1	15/1	80–99	Lambert et al. [136]
Huelva PG stack	Spain	0.0345	H_2_SO_4_	0.5 ^a^	25	2–8	20/1	41–58	Cánovas et al. [135]
			HNO_3_	3.0 ^a^				75–86	
Synthetic PG in Florida	USA	1.0	H_2_SO_4_	0.22 ^a^	25	24	50/1	76.9–93.7	Antonick et al. [145]
			H_3_PO_4_					5–85	
Catarinense Carbochemical Industry S/A in Imbituba, Santa Catarina State	Brazil	0.5	H_2_SO_4_	0.6 ^a^	42	1.0	20/1.7	67.8	Lütke et al. [146]
Catarinense Carbochemical Industry S/A in Imbituba, Santa Catarina State	Brazil	0.5	H_2_SO_4_	2.9 ^a^	55	20 min	20/1.7	90	Lütke et al. [147]
Catarinense Carbochemical Industry S/A in Imbituba, Santa Catarina State	Brazil	0.5	Citric acid	3.0 ^a^	80	1.0	20/1	62.0	Lütke et al. [147]
Yunnan Phosphate Chemical Group in Yunnan Province	China	0.02	HCl	1.65 ^a^	25	2.0	10/1	52	Guan et al. [148]
					60			66	
					80			78	
Yunnan Phosphate Chemical Group in Yunnan Province	China	0.02	HNO_3_	1.65 ^a^	30	2.0	10/1	58.5	Zeng et al. [149]
					60			75.9	
					80			83.4	
Abu-Zaabal Company in Cairo	Egypt	0.048	Boric acid	0.5 ^a^	25	20	5/1	17	Gasser et al. [71]
			Malic acid	1.0 ^a^	25	15 min	5/1	17.7	
			Citric acid	1.0 ^a^	60	15 min	5/1	53.3	

Notes: ^a^ represents molar concentration (mol/L); ^b^ represents mass concentration (wt%).

### 3.4. Chemical Products

Over the last few decades, PG’s chemical conversion has emerged as a promising pathway for acquiring economically valuable raw materials. Researchers have employed the Merseburg ammonocarbonation method to transform PG into ammonium sulfate ((NH_4_)_2_SO_4_) and calcium carbonate (CaCO_3_) [115,150]. Ammonium sulfate is a high-quality fertilizer and calcium carbonate is an important industrial raw material. Idboufrade et al. [151] also implemented this technique to transform PG sourced from Morocco to these two products. In the course of the procedure, the pure compounds (NH_4_)_2_SO_4_ and CaCO_3_ were acquired with a molar ratio of *n*(NH_4_OH/PG) = 4 under the condition of 3.5 h, 25 °C, 500 rpm, and 20 mL/min of CO_2_ flow rate. Moreover, a conversion of PG into calcium carbonate and sodium sulfate (Na_2_SO_4_) was performed by Kolokolnikov and Shatov [152] using washing soda (Na_2_CO_3_).

An alternative choice involves transforming PG into calcium carbonate and potassium sulfate (K_2_SO_4_), which hold significant importance in agricultural and industrial sectors. With this aim, Lachehab et al. [153] carried out the chemical conversion of PG into CaCO_3_ and K_2_SO_4_ using KOH and CO_2_. Based on this, Laaboubi et al. [154] devised a novel crystallization route to convert PG into Na_2_SO_4_ and K_2_SO_4_, employing the quaternary reciprocal system Na^+^, K^+^//Cl^−^, SO_4_^2−^-H_2_O. In this sequence, Na_2_SO_4_ was initially generated through the PG transition. Subsequently, Na_2_SO_4_ reacted with KCl to yield glaserite (NaK_3_(SO_4_)_2_). Lastly, the intermediate product glaserite was dissolved in a KCl solution, leading to the formation of K_2_SO_4_.

Converting PG into commercially viable chemical products represents a promising approach for recycling this waste. Nevertheless, secondary raw materials such as CaCO_3_, Na_2_SO_4_, or K_2_SO_4_ frequently lack a cost advantage when compared to similar products already available in the market. To address this issue, it is crucial to recover economically significant substances from PG during the preparation of these by-products.

### 3.5. Applications in Agriculture

**Figure 9:**
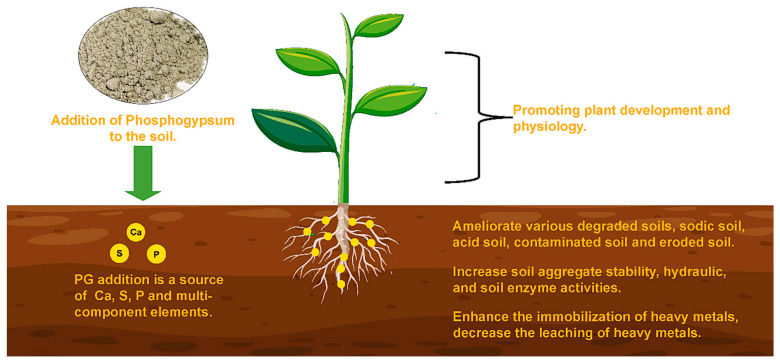
The main advantages of using PG in agriculture [2].

Owing to its acidic property, PG can be used to adjust pH and decrease the salinity of soils. Simultaneously, PG, as a low-grade fertilizer, can supply calcium and sulfur together with traces of P_2_O_5_ [155]. In addition to these benefits, the use of PG in agriculture can also reduce the mobility and availability of some heavy metals in acidic soil [156,157], improve crop yield and soil structure [158], and reduce soil erosion [159,160], as shown in Figure 9.

Nevertheless, the existence of potentially harmful elements, particularly heavy metals and radioactive substances, gives rise to significant apprehensions regarding the utilization of PG as a soil amendment [161]. To ensure the enduring safety of PG application in soil, extensive research has been undertaken to examine the mobility and bioavailability of pollutants in PG. A column leaching study by Nisti et al. [162] involved a blend of typical Brazilian sandy clay soils and PG. They documented a low discharge of metals and radionuclides (such as As, Cd, Ni, Cr, 226-R, 210-Pb, and Th-232) from the soils amended with PG, even at doses ten times greater than those required to attain 50% soil base saturation (approximately 15 g PG/kg soil). Saueia et al. achieved similar findings [163,164]. They assessed the mobility of metals (As, Cd, Cr, Co, Cu, Hg, Ni, Pb, Se, Zn) and radionuclides (Ra-226, Ra-228, and Pb-210) by employing EDTA as the lixiviant. The results indicated that the metals and radionuclides remained inaccessible to the surrounding environment, suggesting the safety of utilizing PG in agricultural practices concerning the potential pollution from such by such elements. Cui et al. [165] conducted a study to investigate the leaching behavior of fluorine (F) in soil. The results revealed that the soil covered with PG released the least amount of F, only 0.18%, implying a minimal impact of PG on F leaching in the soil.

Moreover, some researchers focused on the accumulated impact of impurities in PG on crops [166]. S. Enamorado et al. [167] carried out a research to assess the cumulative effect of trace elements in PG in tomatoes cultivated in soils amended with PG. They discovered that the levels of Cd in tomatoes approached the maximum allowable concentrations, and if PG amendments exceeded 16 g PG/kg soil, it would surpass this limit. Therefore, they suggested that to prevent surpassing legal thresholds for Cd or other contaminants in food, evaluating the content of trace elements contained in PG was imperative before using PG as the soil amendment in agriculture. Similar results obtained by Al-Hwaiti and Al-Khashman [168] reveal that the application of PG as an amendment in soil might lead to concentrations of Cd and Pb in crops exceeding permissible thresholds over an extended period.

In conclusion, leveraging PG in agriculture presents potential advantages because of its ready availability and absence of technological obstacles. Nevertheless, apprehensions stem from impurities contained in PG that might be taken up by plants and, ultimately, pose a threat to human health. Consequently, environmental and public health impacts need to be taken into account when utilizing PG as a soil amendment in agriculture [169].

### 3.6. Other Applications

**Figure 10:**
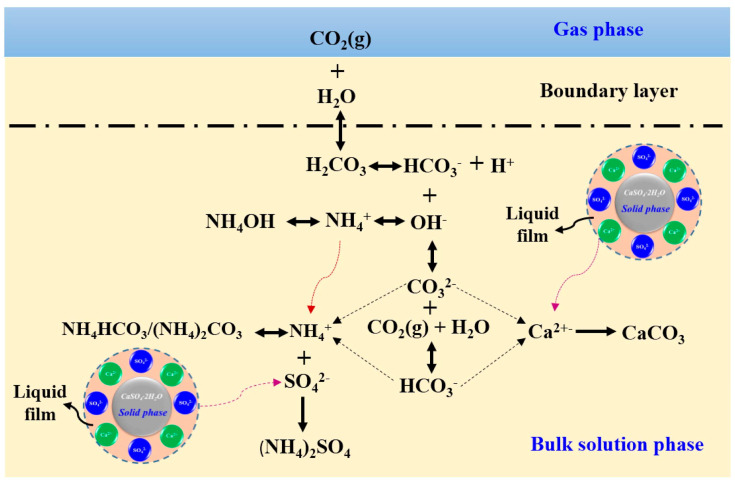
The schematic diagram of CO_2_ sequestration process through PG carbonation using NH_4_OH as an alkaline medium [178].

**Environmental applications:** PG was employed for CO_2_ sequestration through mineral carbonation, demonstrating significant advantages owing to its rapid carbonation rate and high reactivity (>95%) [170,171,172]. Zhao et al. [173] developed an innovative method for storing CO_2_ via the mineral carbonation of PG using NH_4_OH as the alkaline medium under high CO_2_ pressure, during which the highest carbonation conversion of PG was about 97% in 5 min. The mechanism of CO_2_ sequestration process is shown in Figure 10. Moreover, as an economical adsorbent, PG has been employed to eliminate heavy metals from wastewater, with its adsorption capacity predominantly influenced by pH levels [174,175,176,177].

**Papermaking:** Some researchers explored the use of PG as a filler in papermaking. Mechi et al. [179] found that compared to PG, the calcined PG (CPG) at 800 °C showed better performance as a filler in papermaking. The inclusion of CPG significantly enhanced filler retention by up to 97%, leading to remarkable improvements in the optical properties of the filled paper. However, the strength was somewhat adversely affected. In addition, adding the PG whisker to papermaking could significantly improve the index of the paper’s tensile, tearing, bursting, and folding resistance.

**Figure 11:**
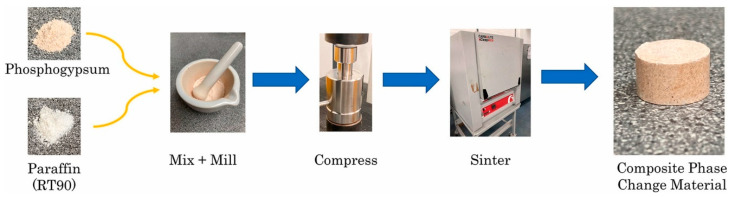
The preparation process of the novel CPCM [182].

**Thermal energy storage material:** Latent heat thermal energy storage technology (LHTES) is an important way to solve the current global energy problems [180]. The fundamental concept of LHTES revolves around a material, often termed as the phase change material (PCM) [181]. The characteristics, such as excellent thermal stability, a substantial surface area, and favorable particle density, position PG as a suitable candidate for supporting materials in PCMs. Anagnostopoulos et al. [182] fabricated a novel composite phase change material (CPCM) consisting of PG and a commercial-grade paraffin (RT90). The fabrication process of the novel CPCM is shown in Figure 11. The novel CPCM exhibited good thermal stability after 96 cycles at 25–100 °C. Its thermal conductivity is notably enhanced to 0.46 W/mK, albeit with a slight decrease in energy storage density to 237.44 MJ/m^3^. This innovative energy product can find application in thermal management and waste heat recovery at low temperatures, a scenario frequently encountered throughout the industrial ecosystem.

All in all, researchers have developed various PG utilization methods in different fields, which have their advantages and limitations in practical applications as shown in Table 8. In fact, large-scale consumption and safe utilization are the major obstacles that need to be addressed during the PG utilization. From solving the two obstacles, utilization of PG in the construction filed, especially using PG as road bases and pavement materials, may be one of the mainstream development directions in the future [183].

## 4. Conclusions

The large accumulation of PG has become a worldwide environmental problem. The impurities in PG and the low-value-added recycling products are the two main barriers to PG utilization. Given these problems, this review paper explored the basic properties of PG and systematically summarized the current purification and utilization techniques, during which the advantages and disadvantages of each technique were discussed in depth.

Among purification technologies, low-cost and easy-to-operate purification technologies, such as washing, cyclone classification, neutralization, and flotation, are more popular in practical production, but these methods cannot effectively remove impurities in an encapsulated state. In addition, methods that can eliminate the intercrystalline impurities are often complex and costly, which hinders their industrial applications. Large-scale consumption and safe utilization are the fundamental ways to solve the current PG accumulation. Based on this, using PG as construction and building materials, especially road bases and pavement materials, may have more excellent development prospects among various PG utilization pathways in the future. Furthermore, preparing high-value-added gypsum products (such as high strength gypsum and calcium sulfate whisker) is an important way to further expand the scale of PG utilization. The traditional high-value path of PG is to remove impurities first and then perform high-value preparation, which is costly and unsustainable. Recently, multiple authors proposed the recrystallization method to efficiently remove impurities from PG and simultaneously produce high-value α-CaSO_4_·0.5H_2_O in one step, as depicted in Section 2.4, which pioneered a sustainable and cost-effective path for a clean and high-value utilization of PG. Additionally, relevant policies and regulations should be improved to actively force enterprises to participate in PG utilization.

## Figures and Tables

**Table 3:** Impurities in PG and their adverse effects [5,12,49,50,51,52].

Impurity	Occurrence States	Effects
Phosphorus	CaHPO_4_·H_2_O, H_3_PO_4_, H_2_PO_4_^−^, HPO_4_^2−^, PO_4_^3−^	Reducing product strength, extending product setting time, accelerating water acidification and water eutrophication
Fluorine	CaF_2_, CaSiF_6_, Na_3_AlF_6_, F^−^	Reducing product strength, entering the environment with rain, entering the human and animal body through the food chain, and causing tooth and bone lesions
Organic matter	Humic substances, humic acid, fulvic acid, extractant agent, foaming agent, defoamer, collector, and ion exchanger	Reducing the whiteness of PG and hindering crystallization
Metals	Rare earth elements	Causing a waste of resources
	Na, Fe, Cr, Cu, Mn	Causing frost or powder phenomenon and heavy metals in products to exceed standards
	Radioactive elements	Damage to the environment and biology
Others	Quartz, black inorganic carbon	Reducing product whiteness

**Table 4:** The main impurity content in the feed and classified products.

No.	*w* (P_total_)	*w* (F_total_)	*w* (Org.)	*w* (Al_2_O_3_)	*w* (Fe_2_O_3_)	*w* (SiO_2_)
R	1.03	0.50	0.99	0.25	0.47	4.26
18-D	0.79	0.87	0.60	0.17	0.41	3.43
18-Y	0.97	1.13	0.55	0.36	0.48	3.32
22-D	0.89	1.08	0.30	0.24	0.46	2.79
22-Y	1.31	1.47	0.79	0.71	0.66	5.98

**Table 5:** Overview of the advantages and disadvantages of the purification methods.

Methods		Advantages	Disadvantages
**Physical methods**	Washing	● Simple ● Easy operation	● Producing large amounts of wastewater ● High cost of wastewater treatment ● Low efficiency ● Difficult to remove the impurities in encapsulated state
	Cyclone classification		
	Sieving		
**Chemical methods**	Acid/alkali leaching	● Simple and easy operation ● High efficiency	● High cost of leachate treatment
	Neutralization		● Difficult to eliminate the impurities locked in the gypsum crystal
**Flotation**		● Mature technology ● High efficiency ● Low operating cost	● Introduction of new contaminants (flotation reagents) ● Difficult to remove the impurities in encapsulated state
**Recrystallization method**		● Effective elimination of intercrystalline impurities ● Efficient removal of impurities ● Simultaneously producing high-value-added gypsum products	● Need more research and development
**Microbiological method**		● Short treatment time ● No secondary pollution ● Low cost	● Limited yield and rates ● Poor applicability
**Heat treatment**		● High efficiency	● High energy consumption

**Table 6:** Advantages and disadvantages of using PG as a construction and building material [115].

Property	Beneficial Impact	Adverse Impact
Unit weight	√	
Fire resistance (10% PG)	√	
Freeze/thaw resistance	√	
Sulfate resistance	√	
Sulfuric acid resistance	√	
Setting time	√	√
Workability		√
Mechanical strength		√
Abrasion resistance		√
Drying shrinkage		√
Soundness expansion		√
Thermal conductivity		√

**Table 8:** Overview of the advantages and limitations of utilizing PG in different fields.

Fields	Advantages	Limitations
**Construction and building materials**	● Large amount of consumption ● Positive effect on unit weight, fire resistance, freeze/thaw resistance, sulfate and sulfuric acid resistance, and setting time of the matrix	● Negative effect on mechanical strength, abrasion resistance, drying shrinkage, soundness expansion, and thermal conductivity of the matrix ● The presence of radioactive elements that could pose a risk to the nearby environment
**Road bases and pavement materials**	● Large amount of consumption ● Positive effect on the unconfined compressive strength ● Alleviating reflection cracks	● Lack of research on its durability or its enduring influence on soil and water resources
**Recovery of rare earth elements (REEs)**	● Increasing the added value of PG utilization ● Removing impurities for subsequent PG utilization in other fields	● Low concentration of REEs in PG ● Difficult to extract intercrystalline REEs ● High cost for only recovering REEs
**Chemical products**	● Preparation of commercial products with high value ● Production of (NH_4_)_2_SO_4_ or K_2_SO_4_ used as a fertilizer	● The utilization of costly reagents ● Complicated production process
**Agriculture**	● Enhancement of soil structure and crop yield ● Restore sodic soil by calcium amendment ● Control surface sealing and reduce runoff and soil erosion ● Increase P content and control N loss of soil	● Contamination of soil by heavy metals and radionuclides present in PG ● Transfer of contaminants from soils amended with PG to agricultural products and ultimately to humans
**Environmental applications**	● Easy available and low cost ● CO_2_ sequestration: fast carbonation rate and high carbonation reactivity ● Economical material for adsorbing heavy metals from wastewater	● Small amount of CO_2_ sequestration ● High pH dependence of heavy metal absorption
**Papermaking**	● As a filler in papermaking	● Need more research and development
**Thermal energy storage material**	● Good thermal stability ● High thermal conductivity	● Need more research and development
