# Correction: Guan et al. The Impurity Removal and Comprehensive Utilization of Phosphogypsum: A Review. *Materials* 2024, *17*, 2067

**DOI:** 10.3390/ma17225574

**Published:** 2024-11-15

**Authors:** Qingjun Guan, Zhuang Wang, Fujia Zhou, Weijian Yu, Zhigang Yin, Zhenyue Zhang, Ru’an Chi, Juncheng Zhou

**Affiliations:** 1 School of Resource Environment and Safety Engineering, Hunan University of Science and Technology, Xiangtan 411201, China; qshq78@163.com (Z.W.); kunjia2012@foxmail.com (F.Z.); ywjlah@163.com (W.Y.); 2 Hunan Province Key Laboratory of Coal Resources Clean-Utilization and Mine Environment Protection, Xiangtan 411201, China; 3 Lithium Resources and Lithium Materials Key Laboratory of Sichuan Province, Tianqi Lithium Corporation, Chengdu 610213, China; 4 School of Xingfa Mining Engineering, Wuhan Institute of Technology, Wuhan 430073, China; zyzxmu@wit.edu.cn; 5 Hubei Three Gorges Laboratory, Yichang 443007, China; rac@wit.edu.cn; 6 School of Mechatronics Engineering, Chengdu University of Technology, Chengdu 610059, China; 18782532617@163.com

In the original publication [1], Figure 2 is cited from the previous publication, but the citation of the original publication was missed. As a result of the investigation and discussions between the authors and the editorial office, it was decided to add the original publication as a reference.

The newly added reference is:

Ref. [62]: Vega-Garcia, D.; Brito-Parada, P.R.; Cilliers, J.J. Optimising small hydrocyclone design using 3D printing and CFD simulations. *Chem. Eng. J.* **2018**, *350*, 653–659.

With this correction, the order of all subsequent references has been adjusted accordingly. The authors state that the scientific conclusions are unaffected. This correction was approved by the Academic Editor. The original publication has also been updated.

## References

[1] Guan Q., Wang Z., Zhou F., Yu W., Yin Z., Zhang Z., Chi R., Zhou J. (2024). The Impurity Removal and Comprehensive Utilization of Phosphogypsum: A Review. Materials.

